# PAMPs of *Piscirickettsia salmonis* Trigger the Transcription of Genes Involved in Nutritional Immunity in a Salmon Macrophage-Like Cell Line

**DOI:** 10.3389/fimmu.2022.849752

**Published:** 2022-04-14

**Authors:** Danixa Pamela Martínez, Cristian Oliver, Natacha Santibañez, José Leonardo Coronado, Ricardo Oyarzún-Salazar, Ricardo Enriquez, Luis Vargas-Chacoff, Alex Romero

**Affiliations:** ^1^ Laboratorio de Inmunología y estrés de Organismos Acuáticos, Instituto de Patología Animal, Facultad de Ciencias Veterinarias, Universidad Austral de Chile, Valdivia, Chile; ^2^ Laboratorio de Fisiología de peces, Instituto de Ciencias Marinas y Limnológicas, Facultad de Ciencias, Universidad Austral de Chile, Valdivia, Chile; ^3^ Centro Fondap de Investigación de Altas Latitudes (IDEAL), Universidad Austral de Chile, Valdivia, Chile; ^4^ Millennium Institute Biodiversity of Antarctic and Subantarctic Ecosystems, BASE, University Austral of Chile, Valdivia, Chile; ^5^ Centro Fondap Interdisciplinary Center for Aquaculture Research (INCAR), Universidad Austral de Chile, Valdivia, Chile

**Keywords:** nutritional immunology, PAMPs (pathogen associated molecular patterns), *Piscirickettsia salmonis*, *Salmo salar*, transcription

## Abstract

The innate immune system can limit the growth of invading pathogens by depleting micronutrients at a cellular and tissue level. However, it is not known whether nutrient depletion mechanisms discriminate between living pathogens (which require nutrients) and pathogen-associated molecular patterns (PAMPs) (which do not). We stimulated SHK-1 cells with different PAMPs (outer membrane vesicles of *Piscirickettsia salmonis* “OMVs”, protein extract of *P. salmonis* “TP” and lipopolysaccharides of *P. salmonis* “LPS”) isolated from *P. salmonis* and evaluated transcriptional changes in nutritional immunity associated genes. Our experimental treatments were: Control (SHK-1 stimulated with bacterial culture medium), OMVs (SHK-1 stimulated with 1μg of outer membrane vesicles), TP (SHK-1 stimulated with 1μg of total protein extract) and LPS (SHK-1 stimulated with 1μg of lipopolysaccharides). Cells were sampled at 15-, 30-, 60- and 120-minutes post-stimulation. We detected increased transcription of *zip8*, *zip14*, *irp1*, *irp2* and *tfr1* in all three experimental conditions and increased transcription of *dmt1* in cells stimulated with OMVs and TP, but not LPS. Additionally, we observed generally increased transcription of *ireg-1, il-6*, *hamp*, *irp1*, *ft-h* and *ft-m* in all three experimental conditions, but we also detected decreased transcription of these markers in cells stimulated with TP and LPS at specific time points. Our results demonstrate that SHK-1 cells stimulated with *P. salmonis* PAMPs increase transcription of markers involved in the transport, uptake, storage and regulation of micronutrients such as iron, manganese and zinc.

## Introduction

Fish innate and adaptive immune responses have humoral and cellular components (1), which are produced/mature and activated/proliferate in primary lymphoid organs (thymus and head kidney) and secondary (spleen and mucosa-associated lymphoid tissue, MALT), respectively (2). The innate immune system involves non-clonal pattern recognition receptors (PRRs) such as C-type lectin-like receptors, Toll-like receptors, and NOD-like receptors, compared to the adaptive immune system that uses highly specific clonal receptors (T- and B-cell receptors) that are able to recognize antigens and their derived peptides (3). Pathogen Associated Molecular Patterns (PAMPs) bind to these PRRs and trigger signaling cascades that activate defensive mechanisms such as phagocytosis, proteolysis, synthesis of antimicrobial molecules and secretion of pro-inflammatory cytokines like *il-1β*, *tnf-α*, *il-18* and *il-6* (4).

Under inflammatory conditions, the innate immune system can induce several antimicrobial mechanisms, including depleting the micronutrients available to pathogens at the systemic and cellular level (5). This defense mechanism is called nutritional immunity and involves depleting micronutrients such as iron, manganese and zinc from the circulation by sequestering them within the cells (5). Nutritional immunity in fish has been described in *Eleginops maclovinus* (6, 7), *Notothenia coriiceps* (8), *Notothenia rossii* (8) and *Salmo salar* (9), being this latter the most important species in the Chilean aquaculture (10). Outbreaks of infectious diseases in *S. salar* farms cause substantial economic losses in the Chilean aquaculture industry every year. The most prevalent etiological agent of these outbreaks in Chile is the Gram-negative bacterium *Piscirickettsia salmonis*, which causes *Piscirickettsiosis* (11). *P. salmonis* cells are pleomorphic, coccoid in shape, usually found in pairs and have a diameter of approximately 0.5-1.5 μm (12). This organism is classified as a facultative intracellular bacteria because it can also grow in agar medium enriched with L-cysteine and iron (13–17), and can synthesize siderophores under limited iron conditions (18) requiring/using different sources of iron (9, 18, 19).

In marine fish, micronutrients such as iron, manganese and zinc are absorbed by the gills and the gastrointestinal tract, having catalytic, structural, physiological and regulatory functions (20). Therefore, it is not surprising that microorganisms have developed sophisticated mechanisms to sequester these micronutrients and use them to survive (5). Restriction of iron availability has been described as a resistance mechanism to infection against *P. salmonis* in fish such as *E. maclovinus* (6, 7) and *S. salar* (9, 21). Additionally, Pulgar et al. (9) indicate that families of *S. salar* resistant to infection with *P. salmonis* can decrease the iron content in the head kidney at 14 dpi, without changes in intracellular zinc levels. This research suggests that components of the innate immune system such as *hamp* and *il-6* may be regulating the nutritional immunity (22, 23), making it effective and efficient in families of *S. salar* with low susceptibility to *P. salmonis*

Proteins involved in iron uptake in *P. salmonis* have already been identified (9, 18, 24), and those involved in zinc and manganese uptake have been identified for other bacteria (25–28). On the other hand, Martínez et al. (8) reported that LPS modulates the expression of iron-related immune genes in *N. coriiceps* and *N. rossi* but does not affect the plasma iron concentrations (8). This latter research suggests that nutritional immunity may not differentiate between live pathogens (that need micronutrients to establish an infection) and PAMPs (that do not). Even, if nutritional immunity is activated by PAMPs, it is unknown which PAMPs from *P. salmonis* would trigger this immune response. Therefore, the objective of this study was to evaluate the transcriptional activation of markers involved in nutritional immunity using the SHK-1 cell line stimulated with different PAMPs isolated from *P. salmonis*.

## Methods

### P. salmonis LF-89

*P. salmonis* LF-89^T^ (ATCC VR-1361) type strain was grown under standard conditions in AUSTRAL-SRS broth for 5 days at 18°C at 50 rpm (29). The strain identity was confirmed using biochemical procedures, PCR, and 16S rRNA sequencing (30).

### SHK-1 Cell Line

SHK-1 cell line (45^th^ passage) was used in this study. SHK-1 cells were cultured in 75 cm^2^-flasks in Leibovitz’s L-15 medium supplemented with 10% FBS (Gibco BRL), 6 mM L-glutamine (Hyclone Laboratories Inc., UT) and 40 µM 2-mercaptoethanol (Gibco, Invitrogen Laboratories, Grand Island, NY). For experiments, cells were cultured without antibiotics in L-15 medium supplemented with 10% FBS at 20°C in 75 cm^2^ flasks (Costar, Fisher Scientific, Ottawa, ON, Canada). Cells were seeded on 6-well plates at 5 × 10^5^ cells per well for 24 h at 20°C and stimulated with PAMPs purified from *P. salmonis*.

### Outer Membrane Vesicles (OMVs)

30 mL of a minimal liquid medium (MLM) supplemented with 3.18 mM cysteine, 2 mM GlutaMAX™ (Invitrogen), and 0.05 mM ferric chloride was inoculated with 1 mL of logarithmic phase *P. salmonis* culture (equivalent to 1 x 10^9^ bacteria) in AUSTRAL-SRS broth. The culture was incubated for 8 days at 18 °C at 50 rpm until the early stationary phase. OMVs were isolated from the culture supernatant of each strain as described by Oliver et al. (31) with some modifications. Briefly, bacterial cells were isolated *via* two consecutive rounds of low-speed centrifugation at 5000 x g for 10 min at 4°C. The bacterial supernatant containing extracellular products was filtered with 0.45 and 0.22 μm/pore-filters to remove residual cells. Finally, OMVs were isolated using an ExoBacteriaTM OMV isolation kit (System Biosciences) according to the manufacturer’s instructions and stored at -80°C until use. The purity of isolated OMVs was confirmed by electron microscopy.

### Total Protein (TP)

Total protein extract was obtained using the protocol described by Oliver et al. (32). Briefly, *P. salmonis* cells were centrifuged at 5000 g for 10 min at 4°C. The bacterial pellets were washed twice in 1x PBS and recentrifuged under the same conditions. The pellet was resuspended in RIPA+, incubated on ice for 20 min and sonicated three times for 5 s on ice. The pellet was incubated for 30 min at 4°C and centrifuged at 12.000 g for 30 min at 4°C. Total protein was quantified using the BCA Protein Assay Kit (Pierce # 23225) according to the manufacturer’s instructions.

### Lipopolysaccharides (LPS)

LPS extract was obtained using an LPS Extraction Kit (ab239718, abcam, BIOSONDA S.A). Briefly, *P. salmonis* was centrifuged (4000 g for 10 min at 4°C), and the pellet was washed in 1x PBS and recentrifuged under the same conditions. The pellet was resuspended in lysis buffer, incubated on ice and sonicated three times for 20 s while on ice. The pellet was then centrifuged at 2500 g for 10 min at 4°C, and the supernatant was transferred to a 1.5 ml tube and treated with Proteinase K. The lysate was heated at 60°C for 60 min and centrifuged at 2500 g for 10 min at 4°C. The LPS in the supernatant was quantified using a total carbohydrate colorimetric assay kit (ab155891, abcam, BIOSONDA S.A) according to the manufacturer’s instructions.

### *In Vitro* Stimulation

SHK-1 cells were exposed to PAMPs isolated from *P. salmonis*. Our experimental treatments were Control (cells incubated with 1 mL culture medium), OMVs (cell stimulated with 1 µg/mL of OMVs of *P. salmonis*), PT (cell stimulated with 1 µg/mL of total protein of *P. salmonis*) and LPS (cell stimulated with 1 µg/mL of LPS of *P. salmonis*). Each treatment was performed in triplicate and sampled at 15-, 30-, 60- and 120-minutes post-stimulation.

### Total RNA Extraction

Total RNA was extracted from treated SHK-1 cells using an E.Z.N.A.^®^ Total RNA Kit I (Omega) according to the manufacturer’s instructions. The RNA pellets were dissolved in diethylpyrocarbonate water and stored at -80°C. The RNA was then quantified at 260 nm on a NanoDrop spectrophotometer (NanoDrop Technologies^®^). Total RNA (400 ng) was used as a template to synthesize cDNA using an MMLV-RT reverse transcriptase (Promega) and oligo-dT primers (Invitrogen), according to standard procedures (7).

### qPCR Analysis

Reactions were carried out on an AriaMx Real-time PCR System (Agilent). cDNA was quantified at 260 nm on a NanoDrop spectrophotometer (NanoDrop Technologies^®^), diluted to 100 ng, and used as a template for the qPCR with reactive Brilliant SYBRGreen qPCR (Stratagene). Primers were designed for transferrin receptor 1 (*tfr1*), divalent metal transporter 1 (*dmt1*), ferroportin (*ireg1*), hepcidin (*hamp*), ferritin heavy-chain (*ft-h*), ferritin middle-chain (*ft-m*), interleukin-6 (*il-6*), iron regulatory protein 1 (*irp1*), iron regulatory protein 2 (*irp2*), zinc transporter 8 (*zip8*), zinc transporter 14 (*zip14*) and *18s*. Reactions were performed in triplicate, and the total reaction volume of 14 µL (6 µL SYBRGreen, 2 µL cDNA (100 ng), 1.08 µL of primers mix, and 4.92 µL of PCR-grade water). The PCR cycle used was: 95°C for 10 min, followed by 40 cycles at 90°C for 10 s, 60°C for 15 s, and 72°C for 15 s. After each reaction, a melting curve analysis of the amplified products was performed to confirm that only one PCR product was amplified and detected. The comparative Ct method was analyzed expression levels (2^-ΔΔCT^) (33). The data are presented as the fold change in gene expression normalized to an endogenous reference gene and relative to the uninfected fish (Control). The specific primers are listed in **Table 1**, and their efficiencies were calculated using the equation E = 10 ^[-1/slope]^ (36).

**Table 1:** Primer sequences.

Gene	Nucleotide sequences (5`→3`)		PCR product size (bp)	Efficiency (%)	Accesion Number	References
*dmt1*	Fw: CGTCTTTTTCACGGGACAGC	Rv: CGTACATGCATATAAATTGGTGGC	126	113.8	–	This study
*ft-h*	Fw: TCTGAACACAACGACCCACA	Rv: GTCAAACAGGTACTCGGCCA	150	105.9	–	Valenzuela-Muñoz et al. (34)
*ft-m*	Fw: TATCACCACGATTGCGAAGC	Rv: CTCGTCGCTGTTCTCCTTGA	150	109.2	–	Valenzuela-Muñoz et al. (34)
*ireg1*	Fw: ACCACCGTGTAGCCCATTAAA	Rv: TTGATAGCTAGCGGGCAGGA	105	101.8	XM_014173032.1	This study
*hamp*	Fw: GCCGATGCATTTCAGGTTCA	Rv: AATGGCTTTAGTGCTGGCAGG	127	106.9	NM_001140849.1	This study
*irp1*	Fw: TTGAGTCGGCTGTGAGGAAC	Rv: GGTCTGAACGGCACCTCTAC	112	100.5	BT045467.1	This study
*irp2*	Fw: TACCAGAGAGACGGGGTTCC	Rv: ACACCCAGTAGGTAGGGTCC	101	107.1	BT072056.1	This study
*tfr*	Fw: GGGTCTAACTGGGAAGCAGC	Rv: AACGGAATGAGACGGATGGG	100	119.0	XM_014188394.1	This study
*zip8*	Fw: ATGAACAGGACGGATCGACG	Rv: AGCATTGGCTCTAACCCAGG	135	87.4	–	This study
*zip14*	Fw: TCCCCATGAACTGGGAGACT	Rv: CAGGATGCCAAAACCCATGC	121	87.2	XM_014143440.1	This study
*il-6*	Fw: GAGCTACGTAACTTCCTGGTTGAC	Rv: GCAAGTTTCTACTCCAGGCCTGAT	129	99.5	XM_014143031.1	Martinez et al. (35)
*18s*	Fw: GTCCGGGAAACCAAAGTC	Rv: TTGAGTCAAATTAAGCCGCA	116	101.9	–	Martínez et al. (7)

### Statistical Analyses

Data were checked for normality and homoscedasticity before performing a two-way ANOVA. When necessary, data were logarithmically transformed to fulfil the required conditions for parametric ANOVA. Two-way ANOVA was used with the time and type of stimulus as factors of variance. ANOVA analyses were followed by a Tukey *post hoc* test to identify differences between different groups. Statistically significant differences were determined using a P < 0.05. Different letters indicate statistical differences in the same stimulus at different time points. Symbols (+, *, #) indicate statistical differences between different stimuli (Control, OMVs, PT and LPS).

## Results

### *P. salmonis* PAMPs Modulate the Transcription of Genes Involved in Micronutrient Transport

**Figure 1:**
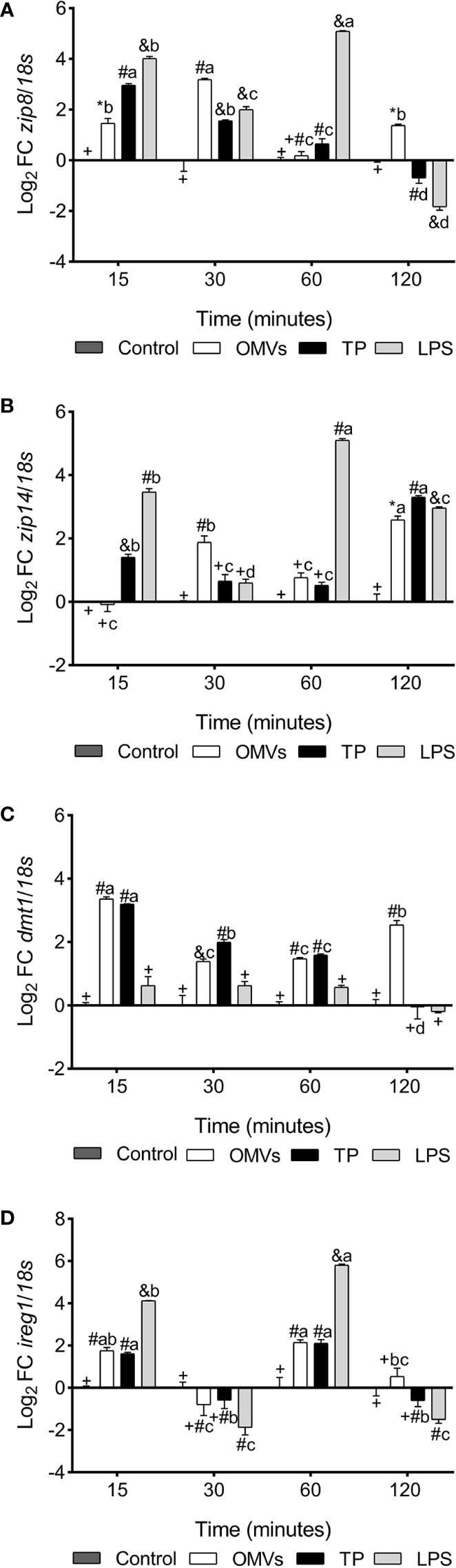
Transcription of *zip8* **(A)**, *zip14* **(B)**, *dmt1* **(C)** and *ireg1* **(D)** in SHK-1 cell line stimulated with 1μg each PAMPs of *P. salmonis* at 15-, 30-, 60- and 120-minutes post-stimulation. Expression analysis of mRNA was performed by qPCR and *18s* was used for normalization. Symbols over the bars indicate statistical differences between the different treatments at the same time points. Different letters indicate statistical differences in the same treatment at different times. Two-way ANOVA, p < 0.05; n=3.

*zip8* transcription was up-regulated at 15-, 30- and 120-min in cells stimulated with OMVs. Similarly, cells stimulated with TP and LPS show an increase in the mRNAs of this gene at 15-, 30- and 60-min, with a statistically significant down-regulation at 120-min (**Figure 1A**).

*zip14* transcription was up-regulated at 30- and 120-min in cells stimulated with OMVs. Similarly, cells stimulated with TP show an increase in the mRNAs of this gene at 15- and 120-min. On the other hand, *zip14* transcription was statistically increased at 15-, 60- and 120-min in LPS-stimulated cells (**Figure 1B**).

*dmt1* transcription was up-regulated at 15-, 30-, 60- and 120-min in cells stimulated with OMVs. Similarly, cells stimulated with TP show an increase in the mRNAs of this gene at 15-, 30- and 60-min. On the other hand, the transcription of this gene did not show statistical differences in cells stimulated with LPS (**Figure 1C**).

*ireg1* transcription was up-regulated at 15- and 60-min in cells stimulated with OMVs and TP. On the other hand, transcription of this gene was statistically increased at 15- and 60-min in LPS-stimulated cells (**Figure 1D**).

### *P. salmonis* PAMPs Modulate Transcription of Genes Involved in Micronutrient Uptake

**Figure 2:**
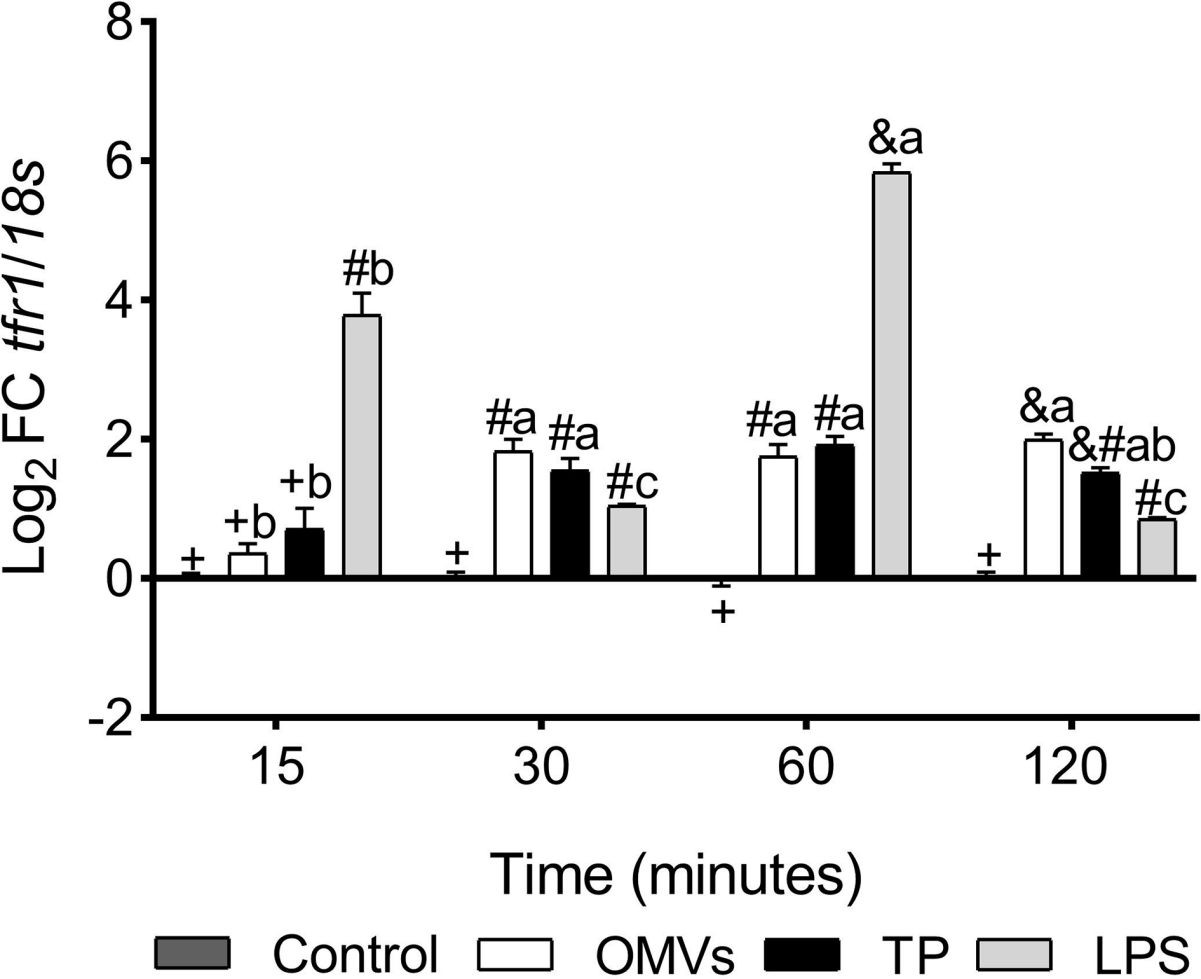
Transcription of *tfr1* in SHK-1 cell line stimulated with 1μg each PAMPs of *P. salmonis* at 15-, 30-, 60- and 120-minutes post-stimulation. Expression analysis of mRNA was performed by qPCR and *18s* was used for normalization. Symbols over the bars indicate statistical differences between the different treatments at the same time points. Different letters indicate statistical differences in the same treatment at different times. Two-way ANOVA, p < 0.05; n=3.

*tfr1* transcription was up-regulated at 30-, 60- and 120-min in cells stimulated with OMVs and TP. On the other hand, the transcription of this gene was statistically increased at 15-, 30-, 60- and 120-min in LPS-stimulated cells (**Figure 2**).

### *P. salmonis* PAMPs Modulate Transcription of Genes Involved in Micronutrient Storage

**Figure 3:**
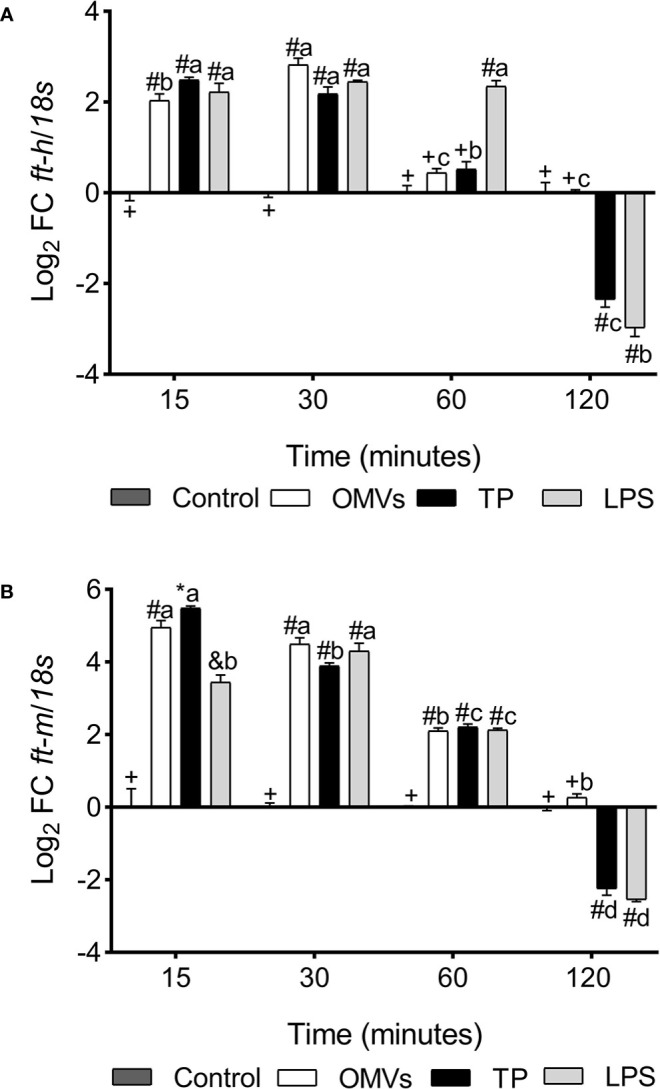
Transcription of *ft-h* **(A)** and *ft-m* **(B)** in SHK-1 cell line stimulated with 1μg each PAMPs of *P. salmonis* at 15-, 30-, 60- and 120-minutes post-stimulation. Expression analysis of mRNA was performed by qPCR and *18s* was used for normalization. Symbols over the bars indicate statistical differences between the different treatments at the same time points. Different letters indicate statistical differences in the same treatment at different times. Two-way ANOVA, p < 0.05; n=3.

*ft-h* transcription was up-regulated at 15- and 30-min in the three experimental conditions. However, at 60-min there was a statistically significant increase only in cells stimulated with LPS, while at 120-min there was a down-regulation in transcription in cells exposed to TP and LPS (**Figure 3A**).

*ft-m* transcription was up-regulated at 15-, 30- and 60-min in the three experimental conditions, while at 120-min there was down-regulation in transcription in cells exposed to treatment with TP and LPS (**Figure 3B**).

### *P. salmonis* PAMPs Modulate the Transcription of Genes Involved in Micronutrient Regulation

**Figure 4:**
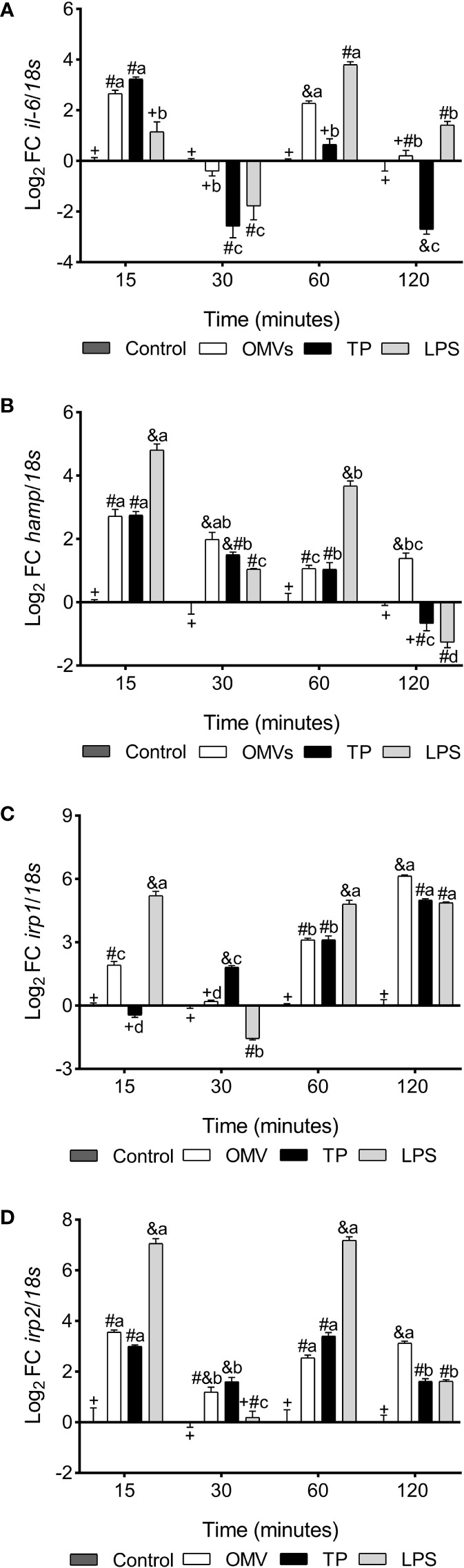
Transcription of *il-6* **(A)**, *hamp* **(B)**, *irp1* **(C)** and *irp2* **(D)** in SHK-1 cell line stimulated with 1μg each PAMPs of *P. salmonis* at 15-, 30-, 60- and 120-minutes post-stimulation. Expression analysis of mRNA was performed by qPCR and *18s* was used for normalization. Symbols over the bars indicate statistical differences between the different treatments at the same time points. Different letters indicate statistical differences in the same treatment at different times. Two-way ANOVA, p < 0.05; n=3.

*il-6* was up-regulated at 15- and 60-min in cells stimulated with OMVs, while cells subjected to TP treatment show up-regulation in transcription at 15-min and down-regulation at 30- and 120-min. On the other hand, cells subjected to LPS treatment decrease the transcription of this cytokine at 30-min, upregulating at 60- and 120-min (**Figure 4A**).

*hamp* was up-regulated at 15-, 30- and 60-min in the three experimental conditions, remaining up-regulated at 120-min in the condition with OMVs and down-regulated at this same time in cells stimulated with LPS (**Figure 4B**).

*irp1* was up-regulated at 15-, 60- and 120-min in cells stimulated with OMVs, while cells stimulated with TP increased transcription of this gene at 30-, 60- and 120-min. On the other hand, LPS treatment increased *irp1* transcription at 15-, 60- and 120- min, statistically decreasing with respect to the control at 30-min (**Figure 4C**).

*irp2* was up-regulated at 15-, 60- and 120-min in the three experimental conditions, while at 30-min it was up-regulated in the treatments with OMVs and PT (**Figure 4D**).

## Discussion

Before this study, it was not known whether nutritional immune responses discriminate between living pathogens (which require nutrients) and non-living pathogens (which do not) or what types of *P. salmonis* PAMPs trigger nutritional immune responses in SHK-1 cells. We observed changes in the transcription of several nutritional immunity associated genes, suggesting that OMVs, TP, and LPS isolated from *P. salmonis* activate nutritional immune responses in SHK-1 cells. The SHK-1 cell line was created using head kidney cells from Atlantic salmon and has macrophage-like characteristics that make it a good model to evaluate the immune response of fish *in vitro*, especially if we consider that *P. salmonis* can modulates the intracellular environment in SHK-1 in the early (vacuolization) and late (propagation) stage of infection to facilitate its survival and propagation (37).

Pulgar et al. (9) showed that *S. salar* families resistant to *P. salmonis* infection had less iron in the head kidney at 14 dpi, but the intracellular zinc levels did not change. We observed increased transcription of *zip8* and *zip14*, which are involved in the uptake of Zn^2+^, Mn^2+^, Fe^2+^ and HSeO_-3_, in all three of our experimental conditions, strongly suggesting that *P. salmonis* OMVs, TP and LPS can alter homeostatic regulation of this micronutrients in SHK-1 cells and increase their uptake into cells. Aditionally, Nebert et al. (38) reported different functions associated to the intracellular increment of these micronutrients by *zip8*, which were related to the immune response, catabolism, oxidative stress, protein glycosylation and cell morphology/proliferation/migration (38). Therefore, the increased *zip8* we observed could suggests an increment in these cellular functions; however, further studies are needed to investigate this directly.

Divalent metal transporter (*dmt1*) is a phagosomal membrane protein that transports iron from the phagosome to the cytosol (39). In our study, the transcription of this transporter was up-regulated in cell line SHK-1 exposed to OMVs and TP, but was not modulated in cells stimulated with LPS. We expected that the three PAMPs used in this study modulated the transcription of this transporter as an antimicrobial response mechanism, since a previous study reported that families of *S. salar* with high susceptibility to *P. salmonis* increases *dmt1* mRNAs in the head kidney to 14 dpi (9). The lack of effects with LPS could be due to a microbial strategy to prevent divalent metals escaping from the phagosomal space to the cytosol, but this should be further investigated for *P. salmonis* in *in vitro* infection assays. When *Francisella* [bacterium similar in terms of pathogenesis to *P. salmonis* (40)] infects, the host cell can induce the synthesis of *dmt1* and ferroportin (*ireg1*), exporting the iron from the phagosome to the cytosol and from the cytosol to the extracellular. However, *Francisella* counteracts this response by inducing a high synthesis of hepcidin (*hamp*) in the host cell, which binds to *ireg-1* and causes its degradation, increasing the iron available in the cytosol for its replication (39).

The modulation of iron homeostasis in macrophages is dependent on the type of pathogen, since *Francisella* uses an active iron acquisition system that is critical for its intracellular proliferation. This system involves the *tfr1* pathway with induction of *steap3*, *irp1*, *irp2* and *dmt1*, while *Salmonella enterica* subsp. enterica *serovar* Typhimurium does not require the expression of these markers for successful intracellular survival (41). We observed increased *irp1*, *irp2* and *tfr1* transcription in all three experimental conditions, suggesting *P. salmonis* acquires iron like *Francisella* does. Our results are consistent with those of Martínez et al. (8), who observed increased *tfr1*, *ireg1* and *hamp* transcription in the head kidney of *N. coriiceps* exposed to LPS. These authors suggest LPS triggers iron associated nutritional immune responses; however, they did not observe any changes in plasma iron concentrations or evaluate tissue iron concentrations.

Iron output is regulated by hepcidin and ferroportin, the union of both proteins causes internalization and degradation of the latter (22). As mentioned previously, in cells infected with *Francisella* the production of *ireg1* is induced, while *Francisella* triggers the synthesis of *hamp* to prevent iron from leaving the extracellular space (39). In our study, the transcription of *ireg1* and *hamp* was modulated in the three experimental conditions. This suggests that the host cell stimulated with OMVs, TP and LPS regulates the synthesis of *ireg1* and that the PAMPs of *P. salmonis*, as in *Francisella*, are sufficient to trigger the transcription of *hamp*. The results are consistent with the observations of Pulgar et al. (9), who reported increased *ireg1* transcription in the head kidney of *S. salar* families with low susceptibility to *P. salmonis* at 14 dpi (9) and for Martínez et al. (7, 8), who reported increased *ireg1* and *hamp* expression in *E. maclovinus* and *N. coriiceps* injected intraperitoneally with two strains of *P. salmonis* and LPS, respectively.

Pro-inflammatory cytokines like *il-6* stimulate *hamp* transcription, triggering and enhancing the hypoferremic response to inflammation (42). We observed increases and decreases in *il-6* transcription in all three experimental conditions, but they did not follow any obvious pattern like those we saw in hepcidin transcription. This can be explained because the levels of mRNAs do not always reflect the levels of protein that are synthesized in the cell and it is likely that in future studies, we will be able to quantify *il-6* in plasma and tissue to have a clearer idea of its relationship with the nutritional immunity. However, *il-6* transcription increases in the kidney and spleen of zebrafish stimulated with OMVs from *P. salmonis*, suggesting OMVs could be candidates for the development of vaccines that provide a protective effect against *P. salmonis* (43). Additionally, *il-6* receptor transcription increases in the head kidney of *N. coriiceps* stimulated with LPS, suggesting a relationship between *il6rβ*, *il-6* and *hamp* (8). Furthermore, others authors have evaluated the nutritional immune responses in *S. salar* (9), and *E. maclovinus* (7) challenged with *P. salmonis*, but they did not measure *il-6* transcription; therefore, it is difficult to compare our results.

*irp1*/*2* proteins modulates iron metabolism in vertebrates by regulating the translation of genes involved in the homeostasis of this micronutrients (44). Under conditions of iron deficiency, *irp1*/*2* bind to the IRE located at the 5’UTR of *ft-h, ft-l* and *ireg1* mRNAs, repressing their translation, while their binding to the IREs of the 3’UTR stabilize the *tfr* and *dmt1* transcripts, preventing their degradation and increasing iron uptake. On the other hand, under overload conditions *irp1/2* decrease their binding activity to the IREs at the 3’UTR and 5’UTR of *tfr*/*dmt1* and *ft-h*/*ft-l*/*ireg1*, respectively. This leads to a destabilization of the *tfr*/*dmt1* mRNAs and an efficient translation of the *ft-h*, *ft-l* and *ireg1* mRNAs, favoring iron sequestration during uptake (44). In our study, the expression profile of *ft-h* and *ft-m* was similar, with an up-regulation in the first minutes of the challenge (OMVs, TP and LPS) and a down-regulation at 120-min in the TP treatments and LPS, suggesting that PAMPs from *P. salmonis* could induce iron storage within the SHK-1 cell line. Our results are consistent with those of Naves et al. (45), who reported a decreased and increased ferritin expression in low and high iron conditions, correspondingly. Additionally, others have reported increased *ft-h*, and *ft-l*/*m* transcription in *N. coriiceps* (8) and *E. maclovinus* (6, 7) exposed to LPS and *P. salmonis*, respectively. However, Pulgar et al. (9) observed increased *ft-l* expression and decreased intracellular iron content in the head kidney of *S. salar* families with low susceptibility to *P. salmonis* at 14 dpi, suggesting increased *ft-l* transcription does not necessarily result in iron storage.

## Conclusion

This study reveals for the first time the temporal expression profiles of markers involved in nutritional immunity in the SHK-1 cell line stimulated with different PAMPs from *P. salmonis*. The results strongly suggest that the three PAMPs of *P. salmonis* used in this study are capable of modulating nutritional immunity in SHK-1, inducing the transcription of immune markers involved in the transport (*zip8*, *zip14*, *ireg1* and *dmt1*), uptake (*tfr1*), storage (*ft-h* and *ft-m*) and regulation (*il-6*, *hamp*, *irp1* and *irp2*) of micronutrients such as iron, manganese and zinc.

## Data Availability Statement

The datasets presented in this study can be found in online repositories. The names of the repository/repositories and accession number(s) can be found below: NCBI, accession IDs: XM_014173032.1, NM_001140849.1, BT045467.1, BT072056.1, XM_014188394.1, XM_014143440.1, XM_014143031.1, AJ427629.1.

## Author Contributions

DM: Writing – original draft, experimental design, sampling, sample analysis, wrote the initial MS version, revision of final MS. CO: Writing – original draft, experimental design, wrote the initial MS version, revision of the final MS. NS and JC: Sample analysis. RO-S: Samples analysis, writing – original draft, revision of the final MS. RE: Maintenance of *P. salmonis* LF-89. LV-C: Writing – original draft, experimental design, revision of the final MS. AR: Writing – original draft, experimental design, revision of the final MS. All authors contributed to the article and approved the submitted version.

## Funding

This work was financially supported by Fondecyt-Postdoctoral N° 3200418, Fondap-Ideal Grant N° 15150003, Fondecyt-Iniciación N° 11180994, Fondap-Incar N° 15110027 and Vicerrectoría de Investigación, Desarrollo y Creación Artística (VIDCA) of the Universidad Austral de Chile.

## Conflict of Interest

The authors declare that the research was conducted in the absence of any commercial or financial relationships that could be construed as a potential conflict of interest.

## Publisher’s Note

All claims expressed in this article are solely those of the authors and do not necessarily represent those of their affiliated organizations, or those of the publisher, the editors and the reviewers. Any product that may be evaluated in this article, or claim that may be made by its manufacturer, is not guaranteed or endorsed by the publisher.
